# Computed tomography and magnetic resonance imaging evaluation of pelvic lymph node metastasis in bladder cancer

**DOI:** 10.1186/s40880-018-0269-0

**Published:** 2018-01-26

**Authors:** Yong Li, Feiyu Diao, Siya Shi, Kaiwen Li, Wangshu Zhu, Shaoxu Wu, Tianxin Lin

**Affiliations:** 1 Department of Radiology, Sun Yat-sen Memorial Hospital, Sun Yat-sen University, Guangzhou, 510120 Guangdong P. R. China; 2 Department of General Surgery, Sun Yat-sen Memorial Hospital, Sun Yat-sen University, Guangzhou, 510120 Guangdong P. R. China; 3 Department of Urology, Sun Yat-sen Memorial Hospital, Sun Yat-sen University, Guangzhou, 510120 Guangdong P. R. China

**Keywords:** Bladder cancer, Lymph node metastasis, Computed tomography, Magnetic resonance imaging

## Abstract

**Background:**

Accurate evaluation of lymph node metastasis in bladder cancer (BCa) is important for disease staging, treatment selection, and prognosis prediction. In this study, we aimed to evaluate the diagnostic accuracy of computed tomography (CT) and magnetic resonance imaging (MRI) for metastatic lymph nodes in BCa and establish criteria of imaging diagnosis.

**Methods:**

We retrospectively assessed the imaging characteristics of 191 BCa patients who underwent radical cystectomy. The data regarding size, shape, density, and diffusion of the lymph nodes on CT and/or MRI were obtained and analyzed using Kruskal–Wallis test and χ^2^ test. The optimal cutoff value for the size of metastatic node was determined using the receiver operating characteristic (ROC) curve analysis.

**Results:**

A total of 184 out of 3317 resected lymph nodes were diagnosed as metastatic lymph nodes. Among 82 imaging-detectable lymph nodes, 51 were confirmed to be positive for metastasis. The detection rate of metastatic nodes increased along with more advanced tumor stage (*P* < 0.001). Once the ratio of short- to long-axis diameter ≤ 0.4 or fatty hilum was observed in lymph nodes on imaging, it indicated non-metastases. Besides, lymph nodes with spiculate or obscure margin or necrosis indicated metastases. Furthermore, the short diameter of 6.8 mm was the optimal threshold to diagnose metastatic lymph node, with the area under ROC curve of 0.815.

**Conclusions:**

The probability of metastatic nodes significantly increased with more advanced T stages. Once lymph nodes are detected on imaging, the characteristic signs should be paid attention to. The short diameter > 6.8 mm may indicate metastatic lymph nodes in BCa.

## Background

Bladder cancer (BCa) is the most common malignant urogenital tumor in China [1] and worldwide [2], although the incidence declined steadily in urban areas in China [1]. Lymphatic spread to the pelvis is the most important route of metastasis of BCa. Once pelvic lymph node metastasis occurs, 5-year survival rate may reduce to 25–35%. Zhang et al. [3] reported that the 5-year cancer-specific survival rate of patients with lymph node metastasis was 27.7%, which was significantly lower than that of patients without lymph node metastasis. If patients with BCa underwent radical cystectomy which included pelvic lymphadenectomy and/or extended lymphadenectomy, the time of operation could be significantly prolonged. Therefore, accurate evaluation of lymph node metastasis in patients with BCa is very important for disease staging, treatment selection, and prognosis prediction [4, 5].

Computed tomography (CT) and magnetic resonance imaging (MRI) have long been used to detect lymph node metastases in patients with BCa before operation. Morphological criteria regarding size and shape are always used for discrimination between benign and malignant lymph nodes [6]. In addition, some new techniques, such as diffusion-weighted (DW) MRI and ultrasmall superparamagnetic iron oxide (USPIO) are used to evaluate the lymph nodes. DW MRI is a noninvasive imaging technique and can be performed with any current MR unit. However, the accuracy of DW MRI in detecting lymph node metastases is still being contested [7]. Although USPIO-enhanced MRI has already shown promising results for the detection of metastases in normal-sized lymph nodes, the limited commercial availability and occasional adverse events restricted its application [8]. Thus the optimal imaging method for detecting metastasis in lymph nodes, especially normal-sized lymph nodes, before surgery is lacking.

The aim of the present study was to retrospectively analyze the diagnostic performance of CT and MRI in the detection of pelvic lymph node metastases comparing with that of pathologic examination in patients with BCa who underwent radical cystectomy and pelvic lymphadenectomy or extended lymphadenectomy.

## Patients and methods

### Patient selection

Institutional Review Board approval was obtained from the Sun Yat-sen Memorial Hospital, Sun Yat-sen University (Guangzhou, China). Patients with BCa who underwent radical cystectomy between July 2007 and March 2017 at Sun Yat-sen Memorial Hospital were included. The exclusion criteria were as follows: (1) patients who underwent neoadjuvant chemotherapy before surgery; (2) patients who underwent laparoscopic radical cystectomy without standard pelvic lymphadenectomy; (3) patients who underwent imaging in other hospitals; (4) CT or MRI performed more than 10 days before surgery; (5) patients with MRI artifacts. All patients met the following selection criteria were included: (1) a contrast-enhanced pelvic CT or MRI performed before surgery in our hospital, with clear images; (2) the lymph nodes resected were recorded and marked group by group; and (3) the bladder tumors and the metastatic lymph nodes were confirmed by pathologic examinations.

### Multidetector spiral CT

All patients underwent contrast-enhanced pelvic CT using a 64-detector row CT scanner (Somatom sensation 64, Siemens Medical Systems, Erlangen, Germany). The CT scanning parameters included a tube voltage of 120 kV, 200 effective mAs, a pitch of 0.8, a gantry rotation time of 0.5 s, and a matrix size of 512  ×  512. After unenhanced CT scanning, dynamic contrast-enhanced CT scanning was performed after intravenous administration of 80–100 mL nonionic contrast material (iopamidol, 370 mg I/mL, Bracco, Milan, Italy) using a bolus-tracking technique at a rate of 4 mL/s and a flush using 20 mL saline. Arterial phase, vein phase, and delayed phase scans were obtained at 25, 60 s, and 3–5 min, respectively. The slice thickness used to reconstruct images for retrospective review was 1.0 mm.

### MRI

MRI examinations were performed with 3.0T superconducting scanner (Philips Achieva, Philips Medical System, Best, the Netherlands) using a phased-array coil. T2-weighted MRI sequence (repetition time/echo time = 2497–3500/70–90 ms; slice thickness, 4.0 mm; number of acquisitions, 2–4) and T1-weighted MRI sequence (repetition time/echo time = 272–497/6–14 ms; section thickness, 3.0 mm; number of acquisitions, 2 or 3) were used. All patients underwent contrast-enhanced scanning after administration of gadopentetate dimeglumine (Magnevist; Guangzhou Schering, Guangzhou, China) at a dosage of 0.1 mmol/kg. Pelvic transverse diffusion-weighted (DW) imaging was performed when necessary. The imaging parameters for DW imaging were as follows: repetition time/echo time/inversion time = 2103/60/180 ms; *b* values, 0 and 800 s/mm^2^; slice thickness, 4.0 mm without gap; sense reduction factor, 2; number of average, 8; echo train length, 31; flip angle, 90°; fast imaging mode, echo-planar imaging; shot mode, single-shot; fat-suppressed mode, spectral presaturation inversion recovery.

### Image analysis and data measurement

Two radiologists with more than 10 years of experience in reading pelvic images reviewed all images without the knowledge of pathologic results. Images were reviewed on Picture Archiving and Communication System (PACS), and determinations were made jointly by consensus. The pelvic lymph nodes were divided into groups according to the drainage region: (1) the perivesical lymph nodes; (2) the bilateral internal iliac, external iliac, and obturator lymph nodes; (3) the presacral, presciatic, and common iliac lymph nodes; and (4) the para-aortic and paracaval lymph nodes. The parameters observed on CT included (1) the size (because the lymph node with maximum short-axis diameter < 3.0 mm couldn’t be identified or measured precisely, we just evaluated the lymph nodes with maximum short-axis diameter ≥ 3.0 mm) and morphologic characteristics (including the presence of the fatty hilum of lymph nodes, the ratio of short/long-axis diameter of lymph nodes, and the margin) of the lymph nodes; (2) the enhancement degree of lymph nodes (CT attenuation value increased 10–40 Hu after enhancement indicates mild-moderate enhancement, and an increase of more than 40 Hu indicates remarkable enhancement); and (3) the presence of necrosis inside lymph nodes. Correspondingly, we observed the following characteristics of MR images: (1) the size (only the lymph nodes with maximum short-axis diameter ≥ 3.0 mm were evaluated) and morphology of lymph nodes; (2) whether the diffusion of the LNs was limited in DW MRI (we did not get a quantitative apparent diffusion coefficient map because many lymph nodes were too small to measure the region of interest); and (3) the enhancement degree of lymph nodes (an enhancement degree similar to that of the bladder muscle layer indicates remarkable enhancement and a lower degree indicates mild-moderate enhancement) and necrosis in lymph nodes.

### Pathologic analysis

The resected tumors and lymph nodes were sampled for conventional hematoxylin–eosin (HE) staining and immunohistological staining for cytokeratin 7 (CK7), cytokeratin 20 (CK20), epithelial membrane antigen (EMA), P63, P53, Vimentin, prostate-specific antigen (PSA), the Ki-67 labeling index, and GATA-binding protein-3 (GATA-3).

### Statistical analysis

The data of CT and MRI were compared with those of pathologic examination. Non-numerical data was analyzed with Kruskal–Wallis test, and categorical data were compared using χ ^2^ test. Differences were considered significant when the *P* values were less than 0.05. Receiver operating characteristic (ROC) curve analysis was performed to evaluate the optimal cutoff value of lymph node size for diagnosing metastasis. The area under the ROC curve (AUC) was evaluated for diagnostic ability. All statistical tests were performed by using the SPSS software (version 20.0; SPSS, Chicago, IL, USA).

## Results

### Patient characteristics

**Fig. 1:**
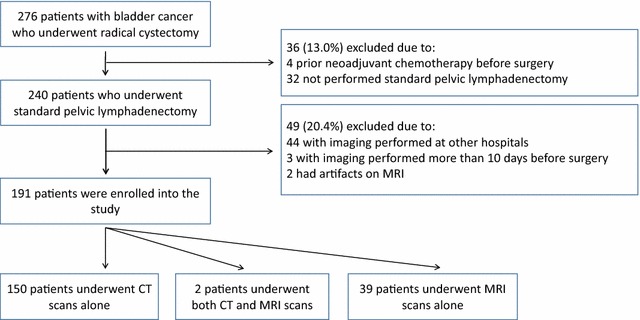
Flowchart of selecting patients with bladder cancer

A total of 276 consecutive patients with BCa underwent radical cystectomy between July 2007 and March 2017 at Sun Yat-sen Memorial Hospital. The following patients were excluded: (1) 4 patients who underwent neoadjuvant chemotherapy; (2) 32 who underwent laparoscopic radical cystectomy but without standard pelvic lymphadenectomy; (3) 44 with imaging performed at other hospitals; (4) 3 with imaging performed more than 10 days before surgery; and (5) 2 with MRI artifacts. Finally, 191 patients were included (Fig. 1).

Of the 191 patients with BCa, 167 were men, and 24 were women. Their median age was 61 years (range, 34–83 years). Most (181) of them had urothelial carcinoma; 47 (24.6%) patients had lymph node metastasis; 150 underwent multidetector spiral CT scan alone, 39 underwent MRI scan alone, and 2 underwent both examinations (Table 1).

**Table 1:** Baseline demographic and clinical characteristics of 191 patients with bladder cancer who underwent radical cystectomy

Characteristic	No. of patients	Percentage (%)
Sex		
Man	167	87.4
Woman	24	12.6
Imaging		
CT alone	150	78.5
MRI alone	39	20.4
Both CT and MRI	2	1.0
Clinical presentation		
Gross hematuria	185	96.9
Microscopic hematuria	6	3.1
Histological classification		
Neuroendocrine carcinoma	3	1.6
Squamous cell carcinoma	3	1.6
Adenocarcinoma	2	1.0
Mucinous adenocarcinoma	1	0.5
Signet ring cell carcinoma	1	0.5
Urothelial carcinoma	181	94.8
Metastasis of lymph nodes	47	24.6

*CT* computed tomography, *MRI* magnetic resonance imaging

### Distribution of lymph node metastasis

A total of 3317 lymph nodes were analyzed by pathologic examination. The median total number of lymph nodes which dissected from each patient was 16 (range, 3–48). Of the 3317 lymph nodes, 184 (5.5%) were diagnosed as pathologic metastasis. The maximum number of metastatic lymph nodes in one patient was 18, and the minimum was 1. The distribution of metastatic lymph nodes according to the drainage region was as follows: 65 (35.3%) perivesical lymph nodes, 22 (12.0%) internal iliac and 56 (30.4%) external iliac lymph nodes, 38 (20.7%) obturator lymph nodes, and 3 (1.6%) presacral lymph nodes, while the presciatic, common iliac, para-aortic, and paracaval lymph nodes were all benign.

A total of 82 lymph nodes were found on CT or MR images, of which 51 were proved to be metastatic lymph nodes by pathologic analysis. As shown in Table 2, the lymph node metastasis rate was the lowest (3.6%) in patients with T1 disease and the highest (75.0%) in patients with T4 disease, with significant difference among all stages (*P* < 0.001). Furthermore, the average numbers of metastatic lymph nodes in one patient also differed significantly across stages (*P* < 0.001) (T1, 0.05; T2, 0.25; T3, 1.75; T4, 4.69); the higher the stage, the more metastatic lymph nodes diagnosed pathologically. A similar trend was observed in imaging results (*P* < 0.001).

**Table 2:** Distribution of metastatic lymph nodes in 191 patients with bladder cancer at different T stages

Final T stage	All patients (No.)	Patients with lymph node metastasis [No. (%)]	Pathological diagnosis		Imaging diagnosis	
			All lymph nodes removed (No.)	Metastatic lymph nodes [No. (%)]	All lymph nodes displayed (No.)	Metastatic lymph nodes [No. (%)]
Total	191	47 (24.6)	3317	184 (5.5)	82	51 (62.2)
pT1	56	2 (3.6)	982	3 (0.3)	6	2 (33.3)
pT2a	32	4 (12.5)	528	6 (1.1)	12	2 (16.7)
pT2b	36	6 (16.7)	633	11 (1.7)	23	10 (43.5)
pT3a	32	13 (40.6)	534	66 (12.4)	12	10 (83.3)
pT3b	19	10 (52.6)	339	23 (6.8)	19	17 (89.5)
pT4a	15	11 (73.3)	281	73 (26.0)	8	8 (100.0)
pT4b	1	1 (100.0)	20	2 (10.0)	2	2 (100.0)

### Imaging characteristics of lymph nodes

**Fig. 2:**
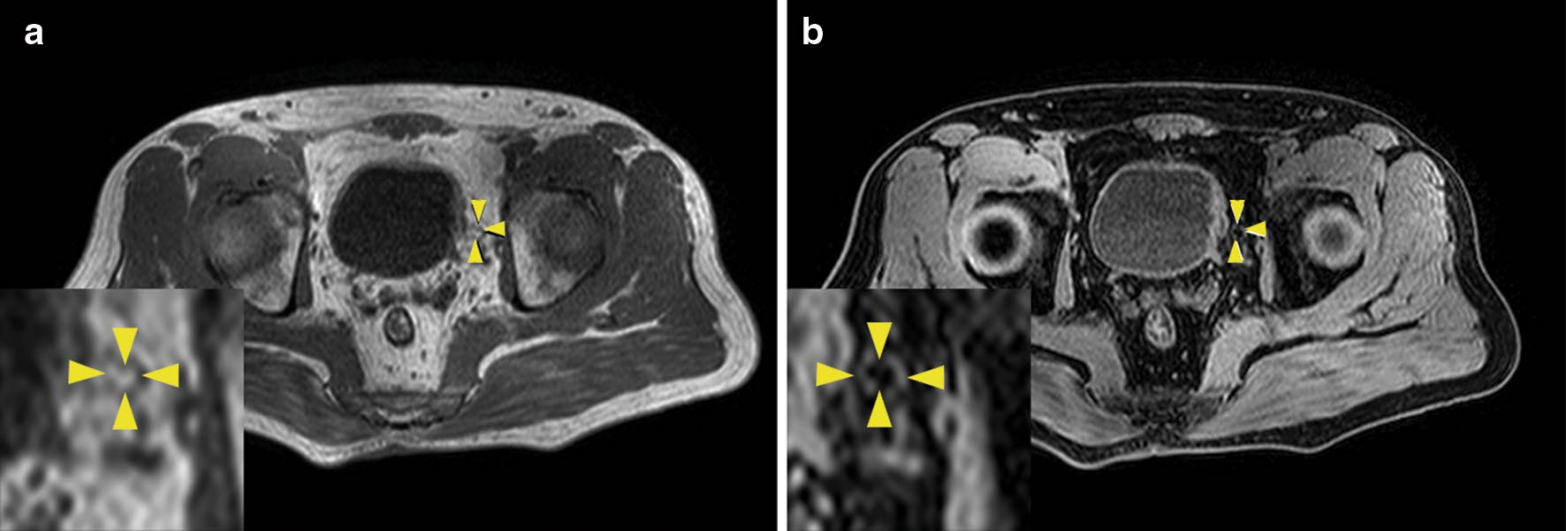
Pelvic magnetic resonance (MR) images of a 62-year-old man with bladder cancer (stage pT2b). **a** Transverse T1-weighted MR image shows a hyperintense lymph node with round shape and a short-axis diameter of about 6.0 mm in the left perivesical region. The fatty hilum shows high signal intensity on the image (yellow arrows). **b** In out-of-phase MR imaging, the fat signal was suppressed in the lymph node (yellow arrows). This lymph node with fatty hilum was proved to be benign in pathologic diagnosis

**Fig. 3:**
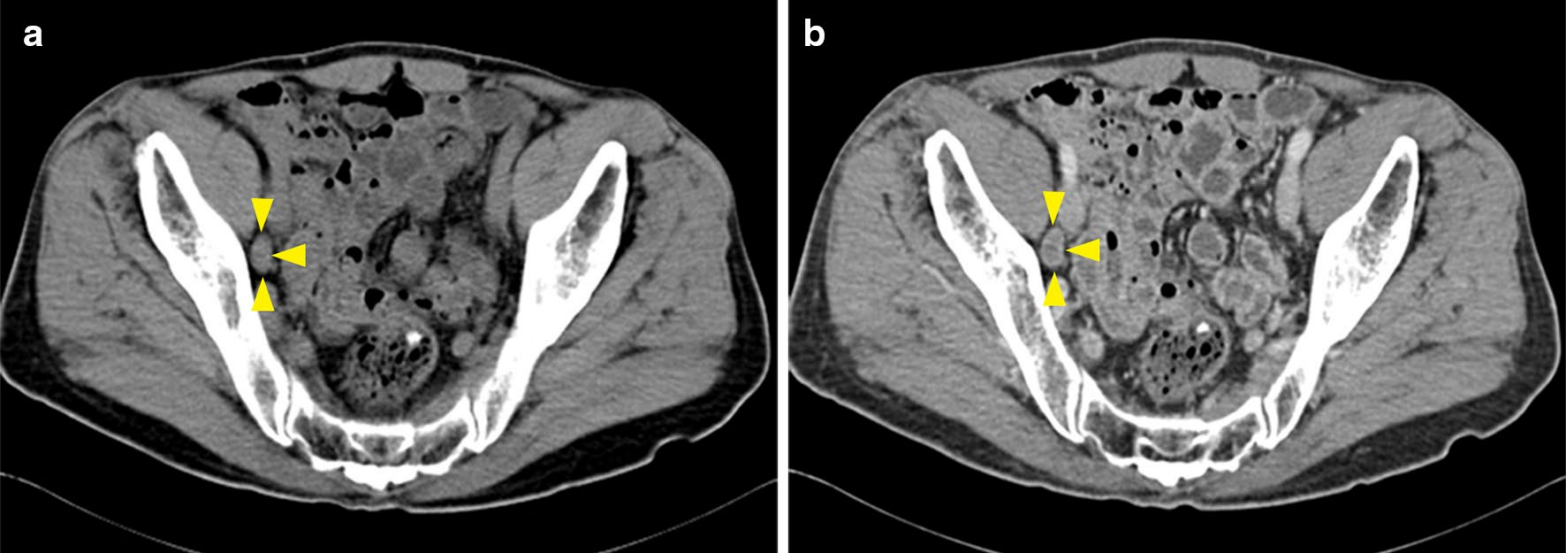
Pelvic computed tomography (CT) images of a 76-year-old man with bladder cancer (stage pT3a). **a** CT scan before contrast shows an isodensity oval lymph node with a short-axis diameter of 9.0 mm in the right obturator region (yellow arrows). **b** In the vein phase after contrast, the necrosis in the center of the lymph node shows annular enhancement (yellow arrows). This lymph node with necrosis was proved to be metastatic in pathologic diagnosis

Of the 3317 resected lymph nodes, only 82 (2.5%) were detected by imaging. However, among the 184 metastatic lymph nodes, 51 (27.7%) were detected by imaging. Three patients had 6–10 metastatic perivesical lymph nodes, but only one of them showed restricted diffusion on DW MRI. Thirty-four metastatic lymph nodes showed focal cancer nest in pathology, but were not detected on images. Lymph nodes shown on images were mainly located in the bilateral iliac, external iliac, and obturator regions. The average short-axis diameter of metastatic lymph nodes was significantly longer than that of non-metastatic lymph nodes (8.6 mm vs. 6.0 mm, *P* = 0.002). According to the short-axis diameter (*d*), lymph nodes were divided into four groups: *d* ≥ 10.0 mm, 8.0 mm ≤ *d* < 10.0 mm, 5.0 mm ≤ *d* < 8.0 mm, and 3.0 mm ≤ *d* < 5.0 mm. As shown in Table 3, a total of 51 lymph nodes among 82 nodes detected on images were positive. When compared with metastatic lymph nodes, more non-metastatic lymph nodes had fatty hilum and the ratio of short- to long-axis diameter ≤ 0.4 (Fig. 2). Meanwhile, more metastatic lymph nodes showed spiculate or obscure margin and necrosis (Fig. 3). However, there was no statistical difference in the degree of enhancement and restricted diffusion on DW MRI between non-metastatic and metastatic lymph nodes. Nine lymph nodes showed both necrosis and annular enhancement on images. Among them, 2 were from 1 patient with mucinous adenocarcinoma, 5 were from 3 patients with squamous cell carcinoma, and 2 were from 2 patients with urothelial carcinoma.

**Table 3:** Characteristics of lymph nodes observed on CT and MRI

Characteristic	No. of lymph nodes		*P* value
	Non-metastatic, (n = 31)	Metastatic, (n = 51)	
Short-axis diameter (d)			0.017
*d* ≥ 10.0 mm	1	15	
8.0 mm ≤ d < 10.0 mm	10	14	
5.0 mm ≤ d < 8.0 mm	11	16	
3.0 mm ≤ d < 5.0 mm	9	6	
Fatty hilum	4	0	0.009
Ratio of short- to long-axis diameter ≤ 0.4	5	0	0.003
Spiculate or obscure margin	0	8	0.021
Necrosis	0	9	0.014
Enhancement degree			0.246
Mild-moderate	13	15	
Remarkable	18	36	
Restricted diffusion on DW MRI	5	7	0.767

*CT* computed tomography, *MRI* magnetic resonance imaging, *DW* diffusion-weighted

**Fig. 4:**
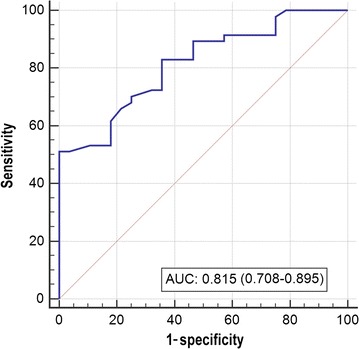
Receiving operating characteristic (ROC) curve of short-axis diameter of metastatic lymph nodes measured on CT and/or MRI (the blue line). A cutoff value of 6.8 mm results in optimal sensitivity (83.0%) and specificity (64.3%) and high Youden index (47.3%). The area under the ROC curve (AUC) is 0.815, and 95% confidence interval is 0.708–0.895

The ROC curve analysis showed that 6.8 mm was the optimal cutoff value of the short-axis diameter for the diagnosis of metastatic lymph nodes, with the AUC of 0.815, the sensitivity of 83.0%, the specificity of 64.3%, and the Youden index of 47.3% (Fig. 4).

## Discussion

The present study showed that the detection rate of metastatic lymph nodes in BCa patients increased with advancing T stage. Lymph nodes with the short-axis diameter of < 3.0 mm were rarely seen on CT and/or MRI. The characteristic imaging signs such as the fatty hilum of lymph node and the ratio of short/long-axis diameter ≤ 0.4 were usually found in non-metastatic lymph nodes, while spiculate margin and necrosis were commonly observed in metastatic lymph nodes. Besides, the cutoff value of short-axis diameter was 6.8 mm in the CT/MR evaluation of lymph node metastasis in patients with BCa.

A previous study reported that the rate of lymph node metastases in patients with BCa who underwent radical cystectomy was about 27% [9]. In the present study, this rate was lower (24.6%), which may be related to the early detection and treatment. With stage advancing, the rate of lymph node metastasis increased gradually. This finding was supported by other studies [10, 11]. The metastatic lymph nodes in patients with BCa in the present study were mainly distributed in the perivesical, internal iliac, external iliac, and obturator regions, with only a few located in the anterior sacral region. The metastatic lymph nodes in the presciatic, common iliac, para-aortic, and paracaval regions were always visible on images in patients with T2–T4 stage disease, suggesting that pelvic lymphadenectomy is necessary for patients with T2–T4 stage diseases. However, extended lymphadenectomy is unnecessary unless the imaging indicated the presence of metastatic nodes outside the presciatic, common iliac, para-aortic, and paracaval regions. This finding is inconsistent with previous findings which recommended radical cystectomy with pelvic lymphadenectomy [12], and further study may be required.

Multidetector spiral CT and MRI are the most common methods for the diagnosis and staging of BCa. In the present study, compared with pathology, imaging detected very few lymph nodes; however, the proportion of metastatic lymph nodes among all lymph nodes detected by imaging was significantly higher than that by pathology (62.2% vs. 5.5%). These results indicate that most of the negative lymph nodes detected by pathology cannot be detected by imaging. Usually, lymph nodes observed on images show varying degrees of reactive hyperplasia or metastasis confirmed by pathology [13]. The number of metastatic lymph nodes detected on imaging was 27.7% of the lymph nodes verified metastases by pathology, which indicated metastatic lymph nodes detected on imaging was much fewer than by pathology. It may due to two main reasons. First, many metastatic lymph nodes contained focal cancer nest or were micrometastasis in pathologic diagnosis, and they were too small to display on image. Second, a lot of metastatic lymph nodes verified by pathology were from the perivesical region, but the perivesical lymph nodes detected on imaging were few which led to huge difference between imaging findings and pathologic results. Some studies indicated that perivesical lymph nodes were dissected in many patients after radical operation with pelvic lymphadenectomy, and the presence of positive perivesical lymph nodes was independently associated with poor survival [14]. Therefore, some imaging characteristics, such as spiculate or obscure margin and necrosis, should receive particular attention. Although it is hypothesized that lymphatic drainage was started from the perivesical region, metastatic lymph nodes in the external iliac and obturator regions were visible while the lymph nodes in the perivesical region were invisible in some patients in the present study. This phenomenon may be attributed to the small size of perivesical lymph nodes or the presence of venous plexus in the perivesical region. Mir et al. [15] showed that the combination of DW and T2W imaging was more sensitive to detect pelvic lymph nodes (167 lymph nodes detected) than T2W imaging alone (114 lymph nodes detected). This result was supported by some studies [16–18], but was inconsistent with the results in the present study and other studies [19, 20]. In future, a larger sample size is needed to test this result with MRI.

With the development of technology, the resolution of both CT and MRI is getting higher and higher. Therefore, the lymph nodes with diameters > 3.0 mm can be easily displayed on images. The small intestine without inflation and small veins could be distinguished from lymph nodes easily. However, not all enlarged lymph nodes are metastatic. The lymph node enlargement may due to fat hyperplasia, sinus tissue cell proliferation, lymphocyte proliferation, and other reasons [5]. We found that some imaging characteristics can indicate the benign changes of lymph nodes, such as the ratio of short/long-axis diameter of lymph node ≤ 0.4 and the presence of fatty hilum, as found by other studies [21, 22]. Some characteristics directly suggest lymph node metastasis, such as spiculate or obscure margin and necrosis [23]. These findings can be used in preliminary screening, but the proportion of lymph nodes with these characteristics is low. Characteristics that appear frequently, such as enhancement degree and signals that indicate restricted diffusion on DW MRI, are not specific. Currently, the size of lymph nodes becomes an important index. In general, the smaller threshold indicates the higher sensitivity and the lower specificity; the larger threshold indicates the lower sensitivity and the higher specificity [4]. However, a cutoff value of 10 mm for the short-axis diameter of metastatic lymph node is not appropriate [24, 25]. The ROC curve analysis in the present study showed that a cutoff value of 6.8 mm for the short-axis diameter of lymph node was optimal for the diagnosis of lymph node metastasis in BCa patients.

There are some limitations in the present study. First, the relationship between the size of primary tumor and lymph node metastasis had not been statistically analyzed. Second, the size of the metastatic foci of lymph node had not been measured in pathologic examination, and thus could not be compared with the imaging data. Third, no statistical analysis was performed to explore the relationship between the degree of differentiation of the primary tumor and metastatic lymph nodes.

## Conclusions

According to the present study, imaging characteristics were in great importance for diagnosing lymph node metastases in BCa patients. A short-axis diameter of 6.8 mm was an optimal threshold for diagnosing metastatic lymph nodes. In the near future, we intend to design a prospective study to explore the relationship between the size of pathologically confirmed metastatic lymph nodes and imaging characteristics. In addition, a validation dataset will be used for our further study.
